# Supplementary material for: Effectiveness and Safety of Ayurvedic Medicines in Type 2 Diabetes Mellitus Management: A Systematic Review and Meta-Analysis
Source: Front Pharmacol. 2022 Jun 8;13:821810. doi: 10.3389/fphar.2022.821810 (PMC9213670; doi:10.3389/fphar.2022.821810)
Supplement: Supplementary file 1 [file DataSheet1.zip › Supplementary Material/Forest and Funnel Plots/Trigonella foenum-graecum/BMI.pdf]

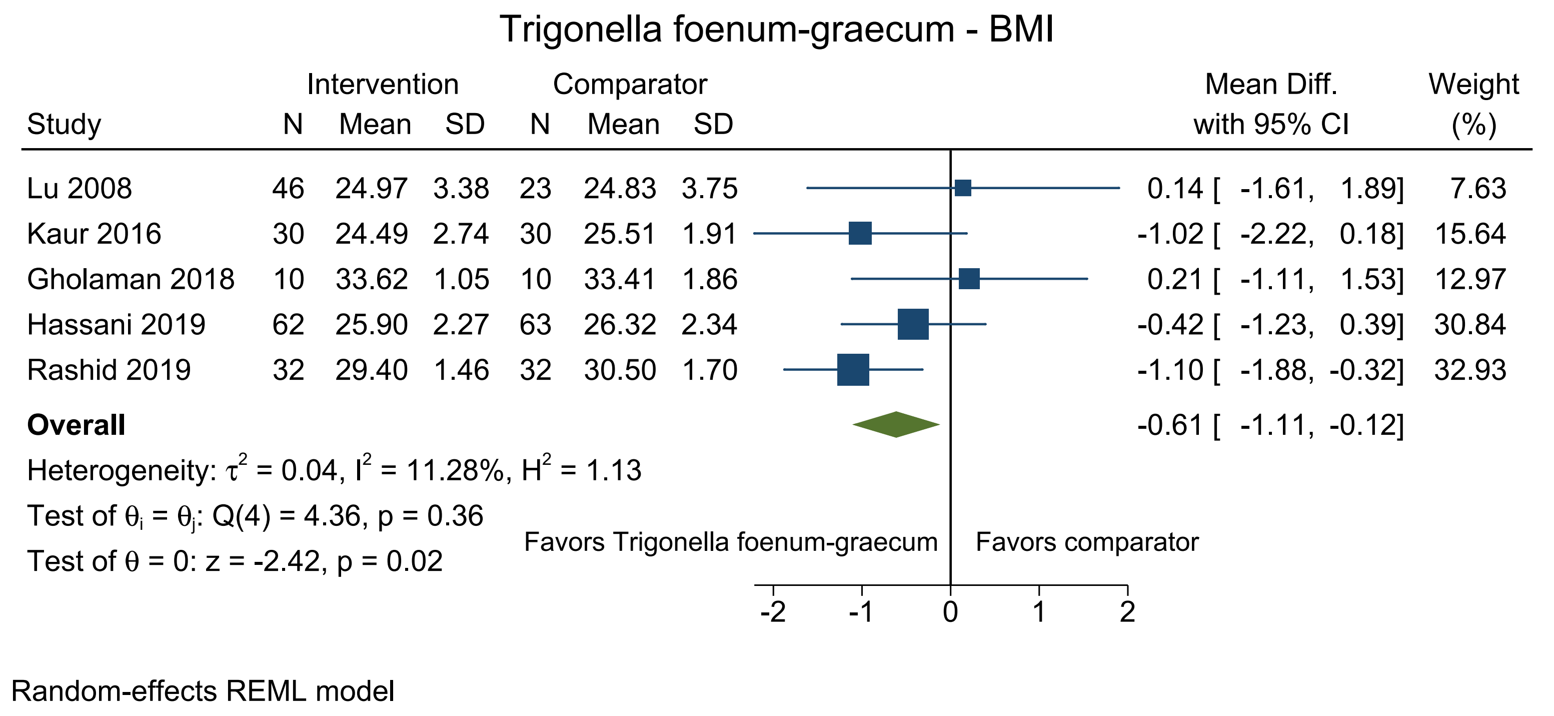
Trigonella foenum-graecum - BMI
Intervention
Comparator
Study
N
Mean
SD
N
Mean
SD
Mean Diff. with 95% CI
Weight (%)
Lu 2008 46 24.97 3.38 23 24.83 3.75 0.14 [-1.61, 1.89] 7.63
Kaur 2016 30 24.49 2.74 30 25.51 1.91 -1.02 [-2.22, 0.18] 15.64
Gholaman 2018 10 33.62 1.05 10 33.41 1.86 0.21 [-1.11, 1.53] 12.97
Hassani 2019 62 25.90 2.27 63 26.32 2.34 -0.42 [-1.23, 0.39] 30.84
Rashid 2019 32 29.40 1.46 32 30.50 1.70 -1.10 [-1.88, -0.32] 32.93
Overall -0.61 [-1.11, -0.12]
Heterogeneity: τ^2 = 0.04, I^2 = 11.28%, H^2 = 1.13
Test of $\theta_i = \theta_j$: Q(4) = 4.36, p = 0.36
Test of θ = 0: z = -2.42, p = 0.02
Favors Trigonella foenum-graecum
Favors comparator
-2
-1
0
1
2
Random-effects REML model
